# IMPACT OF PERCUTANEOUS ENDOSCOPIC GASTROSTOMY ON THE ESOPHAGEAL/GASTRIC MUCOSA AND *HELICOBACTER PYLORI* IN INDIVIDUALS WITH NEUROGENIC OROPHARYNGEAL DYSPHAGIA

**DOI:** 10.1590/S0004-2803.24612026-034

**Published:** 2026-09-28

**Authors:** João Paulo Galletti PILON, Rodolfo de Oliveira MEDEIROS, Paula Cristina COLA, Suely Mayumi Motonaga ONOFRI

**Affiliations:** 1 Universidade Estadual Paulista (UNESP), Faculdade de Filosofia e Ciências, Departamento de Fonoaudiologia, Marília, SP, Brasil.; 2 Universidade de Marília (UNIMAR), Marília, SP, Brasil.

**Keywords:** Swallowing disorders, percutaneous endoscopic gastrostomy, helicobacter pylori, deglutition disorders, esophagitis, Transtornos da deglutição, gastrostomia endoscópica percutânea, helicobacter pylori, disfagia, esofagite

## Abstract

**Background:**

Oropharyngeal dysphagia is frequent among individuals with neurological disorders and may impair nutrition and quality of life. Long-term nasoenteric tube use is associated with esophagitis, gastritis, and increased *Helicobacter pylori*, highlighting the need for safer nutritional alternatives. Percutaneous endoscopic gastrostomy (PEG) is widely used for long-term enteral feeding.

**Objective:**

To evaluate esophageal and gastric inflammatory changes and the presence of *H. pylori* before and 6 months after PEG placement in patients with neurogenic oropharyngeal dysphagia.

**Materials and Methods:**

In this prospective study, 42 patients (median age 76 years) underwent upper gastrointestinal endoscopy and rapid urease testing at baseline and six months after PEG placement. Esophagitis and gastritis were classified using the Los Angeles and Sydney systems. McNemar test was applied (*P*≤0.05).

**Results:**

Esophagitis decreased from 57% to 5% and gastritis from 52% to 10% (both *P*<0.001). *H. pylori* positivity declined from 24% to 12% (*P*=0.154).

**Conclusion:**

PEG was associated with significant improvement in esophageal and gastric mucosal integrity, supporting its role as a safe long-term nutritional strategy in patients with neurogenic oropharyngeal dysphagia.

## HIGHLIGHTS

• Percutaneous endoscopic gastrostomy significantly reduced esophagitis and gastritis in patients with neurogenic oropharyngeal dysphagia.

• Six months after PEG placement, esophageal and gastric mucosal integrity improved substantially.

• *Helicobacter pylori* positivity decreased after PEG, although the reduction was not statistically significant.

• PEG represents a safe long-term enteral feeding strategy with additional benefits for upper gastrointestinal mucosal health.

## RESUMO

**Contexto:**

A disfagia orofaríngea é frequente em indivíduos com doenças neurológicas e pode comprometer o estado nutricional e a qualidade de vida. O uso prolongado de sondas nasoentéricas está associado à esofagite, gastrite e ao aumento da infecção por Helicobacter pylori, ressaltando a necessidade de alternativas nutricionais mais seguras. A gastrostomia endoscópica percutânea (GEP) é amplamente utilizada para a terapia nutricional enteral de longa duração.

**Objetivo:**

Avaliar as alterações inflamatórias do esôfago e do estômago, bem como a presença de Helicobacter pylori, antes e seis meses após a realização da gastrostomia endoscópica percutânea em pacientes com disfagia orofaríngea neurogênica.

**Métodos:**

Neste estudo prospectivo, 42 pacientes (idade mediana de 76 anos) foram submetidos à endoscopia digestiva alta e ao teste rápido da urease no momento basal e seis meses após a realização da gastrostomia endoscópica percutânea. A esofagite e a gastrite foram classificadas de acordo com as classificações de Los Angeles e de Sydney, respectivamente. Foi aplicado o teste de McNemar (*P*≤0,05).

**Resultados:**

A frequência de esofagite reduziu de 57% para 5% e a de gastrite de 52% para 10% (ambos *P*<0,001). A positividade para *H. pylori* diminuiu de 24% para 12% (*P*=0,154).

**Conclusão:**

A gastrostomia endoscópica percutânea esteve associada à melhora significativa da integridade da mucosa esofagogástrica, reforçando seu papel como uma estratégia segura de terapia nutricional enteral de longa duração em pacientes com disfagia orofaríngea neurogênica.

## INTRODUCTION

Oropharyngeal dysphagia is a common condition among individuals with neurological disorders and may significantly compromise nutrition, hydration, respiratory function, and overall quality of life^1^. In a previous systematic review, the prevalence of dysphagia varied widely, with rates reported as 8.1 to 80% among stroke patients, 11 to 81% among Parkinson’s Disease patients, and 27 to 30% among traumatic brain injury patients^2^, representing a significant cause of morbidity and increased healthcare costs^3^.

Although nasoenteric tubes are commonly used for alternative feeding, they are associated with several complications, including local inflammation from reflux or mechanical trauma, pharyngeal injuries, tube obstruction, intestinal perforation, and accidental removal^4^ ^,^ ^5^. Additionally, prolonged use has been linked to a higher prevalence of Helicobacter pylori (*H. pylori*), which contributes to chronic gastritis, peptic ulcer disease, and an increased risk of gastric cancer^6^.

The percutaneous endoscopic gastrostomy (PEG) has become the preferred method for long-term enteral nutrition, due to its safety, minimally invasive nature, preservation of gastrointestinal physiology, lower risk of complications, and high effectiveness in maintaining adequate nutritional support^7^.

In a recent study, Muhtaroglu et al. (2023) reported that 96 patients who underwent PEG procedures, comparing those with the initial PEG tube to those who received a tube replacement, showed significantly lower *H. pylori* positivity in the tube replacement group (*P*=0.022), suggesting that enteral nutrition may reduce the incidence of *H. pylori* infection^8^.

Few studies have investigated changes in the esophageal and gastric mucosa and the presence of *H. pylori* in PEG individuals with neurogenic dysphagia^8^. Considering that neurogenic dysphagia is highly prevalent among individuals with neurological diseases and in geriatric populations^9^, this question represents an important area for investigation. We hypothesized that replacing nasogastric/nasoenteric tubes with PEG would reduce the severity of esophagitis and gastritis and decrease *H. pylori* prevalence over 6 months in this specific population.

The early detection of mucosal changes and *H. pylori* infection is essential to optimizing patient care and achieving benefits beyond simple nutritional support. Therefore, this study aimed to evaluate inflammatory changes in the esophagus and stomach and to investigate the presence of *H. pylori* before and 6 months after PEG placement.

## METHODS

Prospective interventional clinical study. After approval by the institution’s Research Ethics Committee (Approval No. 4.765.102), all participants or their legal guardians agreed to and signed the Informed Consent Form. The research was conducted between 2023 and 2024 at the Medical Specialties outpatient clinic, and all procedures were performed at the General Hospital.

A total of 44 individuals with neurogenic oropharyngeal dysphagia and an indication for PEG were included. The cohort comprised 26 men (59%) and 18 women (41%), with a median age of 76 years (range: 25-94 years; SD = 15.2). Neurological etiologies were 20 (48%) neurodegenerative diseases, 19 (45%) stroke, and 3 (7%) other neurological conditions. Two participants died of unrelated causes before the second evaluation and were excluded from the final analysis, which included 42 individuals. Inclusion criteria were age ≥18 years, a stable clinical condition, and a nasogastric (NGT) or nasoenteric (NET) feeding tube. Patients with contraindications to endoscopy or PEG placement were excluded.

We collected clinical and demographic data. Assessments were conducted twice: baseline (Pre-PEG: during PEG placement) and after six months (Post-PEG: during tube replacement) by an experienced gastroenterologist with over 10 years of clinical practice. At the second assessment, the gastroenterologist was blinded to the patients’ previous clinical information to avoid bias during the evaluation. On both occasions, participants underwent upper gastrointestinal endoscopy using Olympus GIF-Q150 (Olympus America Inc., Center Valley, PA, USA) and Pentax EPK-1000 or EPK-3000 systems (Pentax Medical Co., Montvale, NJ, USA), with complete esophagogastric assessment. A rapid urease test for *H. pylori* (Luckmann®) was also performed. Esophageal findings were classified according to the Los Angeles classification^10^, and gastric findings according to the Sydney System^11^.

When Helicobacter pylori was detected, patients received eradication therapy according to current clinical guidelines, using a standard triple regimen consisting of a proton pump inhibitor, clarithromycin, and amoxicillin (or metronidazole in cases of allergy), as recommended by the American College of Gastroenterology^12^. Eradication was not reassessed during follow-up.

PEG procedures were performed by experienced endoscopists using the Gauderer-Ponsky pull technique^13^, with G-flex® or Endovive PEG® kits, under intravenous sedation in a hospital setting. Procedural steps included asepsis of the abdominal wall, local anesthesia with 2% lidocaine, gastric puncture under endoscopic transillumination, guidewire insertion, and externalization of the feeding tube through the abdominal wall. After six months, participants underwent a second, upper gastrointestinal endoscopy, a new urease test, and tube replacement with a balloon-type feeding device.

Clinical confounders were accounted for by stratifying participants according to the use of proton pump inhibitors (PPIs), anticoagulants, or antiplatelet agents, to control for the potential impact of these medications on esophagogastric mucosal integrity.

The McNemar test was used for sample size estimation, assuming an expected reduction in esophagitis from 50% to 10% after PEG placement, with a 5% significance level (α = 0.05) and 80% power (1 − β = 0.80). A paired-samples formula was applied, and the calculation was performed using the OpenEpi tool, yielding a minimum estimated sample size of 34 participants. The McNemar test was used to compare categorical variables, with significance set at *P*≤0.05, using SPSS (v.26).

## RESULTS

The final sample comprised 42 individuals. Thirty-one participants (74%) had been using a feeding tube for more than three months, and 17 (40%) were taking medications, including proton pump inhibitors, anticoagulants, and/or antiplatelet agents. Endoscopic findings and urease test results before and after PEG placement are summarized in Table 1.

**TABLE 1:** Endoscopic findings and urease test results before and after PEG (N=42).

	Pre-PEG N (%)	Post-PEG N (%)	*P*-value*
Esophagitis	24 (57%)	2 (5%)	<0,001
Gastritis	22 (52%)	4 (10%)	<0,001
Positive Urease Test	10 (24%)	5 (12%)	0,154

*McNemar test

Before PEG placement, esophagitis was predominantly mild (Los Angeles grades A or B), with only two cases classified as grade C and none as grade D. Six months after PEG insertion, residual esophagitis was observed in only two individuals, both with grade A lesions. Pre-PEG gastritis was mainly chronic, mild, and non-atrophic. After PEG placement, gastritis persisted in four individuals, all presenting mild, antral-predominant gastritis without activity or atrophy.

Stratified analyses showed significant reductions in esophagitis and gastritis across most subgroups, particularly among men, older adults, patients with neurodegenerative diseases, and those with longer feeding tube duration (Table 2).

**TABLE 2:** Factors associated with the presence of esophagitis and gastritis before and after PEG.

	Pre-PEG Esophagitis (%)	Post-PEG Esophagitis (%)	***P*-value**	Pre-PEG Gastritis (%)	Post-PEG Gastritis (%)	***P*-value**
Women (n=16)	31	6	0.077	44	0	0.003*
Men (n=26)	73	4	<0.001*	58	15	0.002*
≤75 years (n=19)	37	5	0.020	53	5	0.001*
≥76 years (n=23)	74	4	<0.001*	52	13	0.005*
Feeding tube <3 months (n=11)	45	9	0.068	55	0	0.006
Feeding tube >3 months (n=31)	61	3	<0.001*	52	13	0.001*
Stroke (n=19)	47	5	0.004*	53	5	0.001*
Neurodegenerative disease (n=20)	70	5	<0.001*	50	15	0.018

*McNemar test

No other esophageal or gastric lesions, such as polyps, ulcers, or neoplasms, were identified, and no participant required additional intervention. Because proton pump inhibitors, anticoagulants, and antiplatelet agents may affect mucosal healing, a stratified comparison was conducted between medication users and non-users.

As shown in Table 3, no statistically significant differences were observed between users and non-users of these medications regarding esophageal or gastric inflammatory findings, either before or six months after PEG placement. These results suggest that the improvement in mucosal integrity observed in this cohort is primarily related to the change in feeding route rather than to isolated pharmacological effects.

**TABLE 3:** Esophagitis and gastritis before and after PEG according to medication use (N=42).

Condition	Medication users n=17	Non-users n=25	***P*-value**
Esophagitis - Pre-PEG	10 (59%)	14 (56%)	0.78
Esophagitis - Post-PEG	1 (6%)	1 (4%)	0.80
Gastritis - Pre-PEG	9 (53%)	13 (52%)	0.92
Gastritis - Post-PEG	2 (12%)	2 (8%)	0.71

McNemar test

## DISCUSSION

This study demonstrated that PEG significantly improves esophageal and gastric mucosal inflammation in individuals with neurogenic oropharyngeal dysphagia, particularly among elderly patients and men. These results support the hypothesis that replacing nasoenteric tubes with PEG not only optimizes nutritional support but also reduces the adverse effects of prolonged tube feeding on the upper digestive tract. This research is one of the first prospective, longitudinal studies to apply standardized classifications (Los Angeles and Sydney) and conduct pre- and post-PEG evaluations in a neurogenic dysphagia cohort, thereby highlighting the originality of our findings within the existing literature.

Removing the nasoenteric tube, which impairs the function of the lower esophageal sphincter, appears to favor the restoration of the anti-reflux barrier and reduce the incidence of esophagitis, consistent with findings by Gomes Jr et al.^14^ and Ribeiro et al.^15^. The significant reduction in gastritis may be related to decreased continuous mechanical injury, improved gastric transit, and a more balanced microbiota, as described by Gong et al.^16^ and Motta et al.^17^. By demonstrating a decrease not only in the prevalence but also in the severity of inflammatory lesions, our results provide clinical evidence that PEG contributes to mucosal recovery beyond nutritional maintenance.

Among the main positive effects, reversal of esophagitis and gastritis associated with prolonged nasoenteric tube use stands out. These benefits were more pronounced in men, older patients, and those with tube feeding durations exceeding 3 months. Although the *H. pylori* infection rate decreased six months after PEG, this reduction did not reach statistical significance. Nonetheless, this finding aligns with studies suggesting that changes in gastric pH and microbiota composition, resulting from alterations in the feeding route, may influence *H. pylori* colonization^8^ ^,^ ^18^. The persistence of infection in some patients may be related to increasing antimicrobial resistance, as noted by Shao et al.^19^, even after treatment according to current guidelines.

In addition to the pathophysiological benefits described, it is essential to note that early PEG indication may lead to broader improvements in the clinical management of patients with neurogenic dysphagia^20^. Reducing esophagogastric complications not only improves nutritional status but also decreases the need for additional medical interventions, such as hospitalizations for gastrointestinal bleeding or reflux-related decompensations^21^. In this regard, PEG adoption should be understood within a multiprofessional context, involving physicians, nurses, speech therapists, and nutritionists, to enhance comprehensive and humanized patient care^22^.

However, this study has limitations. Our population consisted exclusively of outpatients, limiting the generalizability of the results to hospitalized or critically ill patients. In addition, endoscopic examinations were performed by a single professional, who, despite clinical experience, may have introduced observer bias. The absence of a control group that continued nasoenteric tube use also limits external validity. Nevertheless, the prospective design, standardized classifications, and paired pre- and post-PEG evaluations are strengths that enhance the validity and reliability of our findings.

From a scientific and epidemiological perspective, our findings open avenues for further studies assessing endoscopic parameters and outcomes related to quality of life, the incidence of aspiration pneumonia, length of hospital stay, and healthcare costs 3. Multicenter studies with larger, more diverse samples, including hospitalized populations, and longer follow-up periods could clarify whether the observed benefits in mucosal integrity translate into lower mortality and a positive impact on healthcare systems^23^ ^,^ ^24^. Long-term microbiological follow-up is necessary to validate the sustainability of these benefits and to guide personalized enteral nutrition strategies. Thus, PEG emerges as an individual clinical strategy and a potential public health measure to reduce complications associated with long-term enteral feeding^25^.

## CONCLUSION

PEG had a positive effect on the esophageal and gastric mucosa in individuals with neurogenic oropharyngeal dysphagia, significantly reducing esophagitis and gastritis after six months. Although the decrease in *H. pylori* infection did not reach statistical significance, a trend toward reduction was observed. Endoscopic findings indicated that factors such as age, sex, duration of tube use, and neurological etiology may influence the response to PEG.

## Data Availability

Data-available-upon-request
